# Erie County Medical Society

**Published:** 1883-10

**Authors:** 


### Erie County Medical Society.

SPECIAL MEETING, SEPTEMBER 8, 1883, TO RECEIVE THE REPORT OF THE COMMITTEE ON LEGISLATION, EMBODYING A DRAFT OF "AN ACT TO ESTABLISH THE MEDICAL FACULTY OF THE UNIVERSITY OF THE STATE OF NEW YORK AND TO REGULATE THE LICENSING OF PRACTITIONERS OF PHYSIC AND SURGERY."

In the absence of the President (who was not in the city), Dr. Hopkins, as Vice-President, called the meeting to order, and having stated the object of the meeting to be the reception of the report of the Committee on Legislation, etc., of which committee he had the honor to be Secretary, he therefore asked Dr. Storck to take the Chair. Dr. Hopkins prefaced the reading of the bill by the following remarks:

"The University of the State of New York was intended by its founders to be the organic head of the educational system of the State. Its influence was to be exerted and its control exercised over the various institutions of learning by a direct supervision of their incorporation, and then by a continuous system of visitation and reports. The University was first incorporated in 1784, and its present authority is a law passed in 1787, which law is said to have been drawn by Alexander Hamilton, who was a member of the assembly at that time. The control of the University is by its Board of Regents, which consists of four *ex-officio* and nineteen other members, chosen by the legislature. The *ex-officio* members are the Governor, the Lieutenant-Governor, the Secretary of State, and the Superintendent of Public Instruction. The elected members must be citizens of the State, and must not be officers of any college or academy. The ninety-fifth report of the Regents, for 1882, includes returns from thirty-six colleges, having an attendance of 9,923 students, and showing graduates for the year, 1,644 in number. Besides this, the Regents visit about 250 schools of academic grade and distribute to them a school fund of about $40,000 a year.

"The uniform and intelligent development of the educational interests of the State has not altogether followed the lines laid down by the framers of the constitution of our University, in that the legislature has, from time to time, granted charters to colleges upon terms less likely to secure the permanent efficiency of such institutions than would the terms imposed by the Regents, the policy of the Regents having been to discourage the establishment of feeble and ill-endowed institutions and to foster and encourage those with ample endowments. Although occasional legislative acts have been at variance with the educational policy of the University and of the State, the great mass of legislation is quite in harmony with, and, in fact, has been shaped by that policy.

"As a member of the Committee on Legislation of the Medical Society of Erie County, the writer was led to look with some little care into the history of medical education in our State, and, as a result of such study, is of the opinion that the faults of our present time are in great measure due to the neglect on the part of the proper authorities to develop and carry into effect the profoundly wise and statesmanlike ideas of Hamilton and his associates, shown in the formation of our State University. The bill which that committee is about to report to the county society is drawn with the view of attempting to turn the stream of medical education into the channel indicated by the early framers of our educational policy. This bill proposes the establishment of the Medical Faculty of the University of the State of New York, and further proposes to endow that Faculty with the sole authority to grant licenses to practice physic and surgery in the State. In conversation with members of the committee, it was thought that it would not be without interest to the society to have a brief digest of this bill submitted for its consideration. This bill will probably be offered upon the authority and responsibility of our county society, and it is the hope of the committee that under such authority the co-operation of the medical profession of the State may be secured to urge its passage by the next legislature.

"As before remarked, the bill provides for one more step in carrying out the idea of a University, by providing for the Medical Faculty of such University, and will have the immediate result of separating the teaching and the licensing powers. Under its operation the faculties of the various medical colleges will teach, but that of the University only will license. Not only will the Faculty of the University not teach, but no man will be eligible to a position upon that Faculty who is connected with any teaching Faculty. The divorce of the teaching and licensing powers is to be absolute and unconditional. This is in harmony with the plan of the University, which provides that no person officially connected with any college or academy shall be eligible to the position of Regent.

"The bill provides that the Governor shall appoint this Faculty, consisting of nine members, who shall be legally authorized practitioners of physic and surgery, and stipulates that none of them shall be connected with any medical school, and that, in such appointments, the several systems of medicine shall receive *pro rata* representation, and that no one can enter upon the practice of medicine in this State after November 1, 1884, without the license of this Faculty. Appointments shall be made for three, four, and five years; after the first all shall be made for the long term. This Faculty is made the highest medical body in the State, and is to have the oversight of all medical societies and institutions, and may be appealed to by all such societies upon any question at issue in the same concerning any subject upon which such society is authorized to make by-laws, and is expected to so influence such societies that they may more efficiently carry out the object for which they were established. The proper officer may administer an oath, and the Faculty may take testimony upon any matter coming within their powers. They shall meet at least twice a year, and examine candidates for licenses to practice medicine, and shall report in writing to the Regents of the University all questions, answers, and opinions in each case. The examination shall be in anatomy, physiology, histology, pathology, theory and practice of medicine, chemistry, surgery, obstetrics, materia medica, therapeutics, and such other branches as they may consider proper. The questions in materia medica, therapeutics, practice of physic and surgery or obstetrics, except the practical parts thereof, shall be proposed by an examiner in the same school to which the candidate wishes to be licensed; in all other respects the examinations shall be the same in each case. The records of the examinations and the findings of the Faculty are to be a part of the public records of the University. Graduates of medical colleges in good standing with the Faculty, who are over twenty-one years of age, and of good moral character, are eligible to examination on paying a fee of twenty-five dollars. At least six members must vote in favor of a candidate to give him a license, and then the Regents of the University grant the license, for which the candidate pays the further sum of fifteen dollars. The Faculty also have the power to declare what shall be unprofessional conduct, and to withhold or revoke licenses for the same; such a vote must also be by a two-thirds majority. The Faculty shall elect such officers and establish such rules as they may deem necessary, subject to the approval of the Regents. The bill has no action upon persons now practicing under a legal registration, and provides that all persons wishing to commence the practice of medicine in this State must, besides getting the license of the Medical Faculty of the University, register in the county clerk's office the name, residence, place and date of birth, date of diploma, the institution granting the same, and the date of license to practice in this State; shall exhibit the diploma and license, and swear to the same; to swear falsely in registering will constitute perjury, and to practice under an illegal diploma, or without license, shall be a misdemeanor, rendering the person guilty thereof liable to a fine of two hundred and fifty to five hundred dollars for the first offense, and afterwards to the same fine, and to imprisonment for not less than thirty, nor more than one hundred and eighty days. The bill defines the term 'practice of physic or surgery.' It charges itinerant venders of drugs or nostrums a license fee of one hundred dollars a month, and exempts from its provisions the commissioned medical officers of the United States army, navy, and marine-hospital service, and the members of the house staff of any legally incorporated hospital."

The full text of the bill is as follows:

AN ACT TO ESTABLISH THE MEDICAL FACULTY OF THE UNIVERSITY OF THE STATE OF NEW YORK, TO REGULATE THE LICENSING OF PRACTITIONERS OF PHYSIC AND SURGERY, AND TO FURTHER REGULATE THE PRACTICE OF PHYSIC AND SURGERY.

SECTION 1. On or before the first day of June, 1884, the governor shall appoint the Medical Faculty of the University of the State of New York, to consist of nine (9) members, who shall be legally authorized practitioners of physic and surgery in this State, but none of whom shall be connected with any medical school or college; provided, that in the appointments made the representation of the several systems of medical practice recognized by the incorporated State medical societies of this State shall be in the proportion of six, two and one, that is to say, the system having the largest number of licensed practitioners to have six, that having the next largest to have two, and the remaining system to have one representative; and all persons desiring to enter upon the practice of physic and surgery in this State after November 1, 1884, shall, before so doing, comply with the provisions hereinafter prescribed and obtain the license hereinafter provided.

SEC. 2. Of the nine members of the said Medical Faculty three shall serve, in the first instance, for three years, three for four years, and three for five years; and these terms shall be severally distributed by lot at the first meeting of the said Faculty. All appointments made in the Faculty at the expiration of the several terms fixed above shall be made uniformly for the period of five years each. All vacancies occurring in the said Faculty, from whatever cause, shall be filled, before the next semi-annual meeting of the same, by the appointment by the Regents of the University, from persons nominated by the several State medical societies (in case such nominations shall be made) of a practitioner of the system of practice that had previously been represented by the seat so vacated.

SEC. 3. The said Medical Faculty shall examine all applicants for license to practice physic and surgery in this State. They shall meet at least semi-annually, and at such meetings shall faithfully examine all candidates referred to them for that purpose by the chancellor of said University and furnish him a detailed report in writing of all the questions and answers of each examination, together with a separate opinion of each examiner as to the qualifications and merits of the candidates in each case. The president or secretary of the Faculty shall have authority to administer oaths, and the Faculty to take testimony in all matters relating to its duties.

SEC. 4. Such examination shall be in anatomy, physiology, histology, pathology, theory and practice of medicine, chemistry, surgery, obstetrics, materia medica and therapeutics, and such other branches in the several departments of medical science as the said Faculty may agree upon, subject to the approval of the Regents of the University. The questions forming such examinations shall be the same for each class of candidates offering themselves, with the exception of the departments of materia medica, therapeutics, practice of physic and surgery or obstetrics, except the operative practical parts thereof, in which branches the questions for each candidate shall be prepared by the representatives in the board of examiners, of the system of practice to which such candidate wishes to be licensed.

SEC. 5. The said reports of the examination shall forever be a part of the public records of said University, and the orders of the chancellor addressed to the Medical Faculty, together with the action of the Regents in each case, shall accompany the same.

SEC. 6. Any person, on paying twenty-five dollars into the treasury of the University, and on applying to the chancellor for the aforesaid examination, shall receive an order addressed to the aforesaid Medical Faculty, instructing them to examine the candidates at one of the regular semi-annual examinations, provided that proof satisfactory to the chancellor is first given that the candidate is over twenty-one years of age, of good moral character, and has received a diploma issued to him or her, conferring on him or her the degree of doctor of medicine from some legally incorporated medical college held to be in good standing by the said Medical Faculty.

SEC. 7. The Regents of the University, after finding that not less than two-thirds of the members of said Faculty have voted in favor of a candidate, and that such examination has been a satisfactory test of the qualifications of said candidate, shall issue to him or her a license to practice physic or surgery in the State of New York, for which license the candidate shall pay to the University the further sum of fifteen dollars.

Said Faculty may refuse to recommend a license to individuals guilty of unprofessional or dishonorable conduct, and they may revoke licenses for like cause upon a two-thirds majority vote after giving the accused an opportunity to be heard in his or her defense.

SEC. 8. The moneys paid to the University under the provisions of this act shall be appropriated by said Regents for, and shall defray the expenses incurred under the provisions of this act.

SEC. 9. The Medical Faculty shall establish such rules and regulations and elect such officers as they may deem necessary to insure the faithful execution of this act, subject to the approval of the Regents of the University.

SEC. 10. Subsequent to the first day of November, in the year 1884, every person (excepting such as have heretofore lawfully registered pursuant to the laws of the State in force at the time of the passage of this act) upon complying with sections 6 and 7 of this act shall, before commencing to practice physic or surgery, register in the clerk's office in the county where he or she practices or intends to practice physic or surgery, in a book to be kept by the said clerk, his or her name, residence and place and date of birth, together with the date of his or her diploma and by what institution granted, with his or her license to practice physic or surgery within this State; at the same time the person so registering shall exhibit both the license and the diploma herein required to the clerk, and subscribe and verify by oath or affirmation, before a person duly qualified to administer oaths under the laws of this State, an affidavit containing a plain statement of all the facts as aforesaid, including his or her age, and shall file said affidavit and copy of said diploma with said clerk; said county clerk to receive a fee of twenty-five cents for such registration, payable by the person so registering. Nothing in this section shall be so construed as to prohibit medical consultations in the different counties of the State between legally qualified and registered physicians of this and neighboring States.

SEC. 11. A person who shall willfully swear falsely to any statement contained in the affidavit required by the tenth section of this act shall be deemed guilty of and subject to conviction and punishment for perjury, and a person who violates any of the other provisions of this act, or who shall practice physic or surgery in this State under cover of a diploma unlawfully issued or illegally obtained, or without a license as provided for in this act, shall be deemed guilty of a misdemeanor, and on conviction thereof shall be punished by a fine of not less than two hundred and fifty dollars nor more than five hundred dollars for the first offense, and for each subsequent offense by a fine as aforesaid and by imprisonment for not less than thirty days and not more than six months. The fine when collected shall be paid, one-half to the person or corporation making the complaint, the other half into the county treasury, of the county where such conviction shall be had.

SEC. 12. For the purpose of this act the words "practice physic or surgery" shall mean to annex the letters "M. D." to one's name, or to suggest, recommend or prescribe, direct or employ, as a matter of business or for a fee, for the use of any person, any drug, medicine, appliance, apparatus or other agency, whether material or not material, for the treatment, cure, relief or palliation of any real or supposed ailment or disease of the mind or body, or for the treatment, cure or relief of any wound, fracture or other bodily injury or infirmity, or any deformity. Any itinerant vender of any drug, nostrum, ointment or appliance of any kind, intended for the treatment of disease or injury, or who shall, by writing or printing, or any other method, publicly profess to cure or treat diseases, injuries or deformities by any drug, nostrum, manipulation or other expedient, shall pay to the State a license of one hundred dollars per month, to be collected as provided for by law, as all other licenses are now collected.

SEC. 13. Nothing in this act shall apply to commissioned medical officers of the United States army or navy, or of the United States marine hospital service, nor to any of the members of the house staff of any legally incorporated hospital during the term of their service as such.

SEC. 14. So much of chapter seven hundred and forty-six, laws of eighteen hundred and seventy-two; chapter five hundred and thirteen, laws of eighteen hundred and eighty; chapters eighty-six and six hundred and seventy-nine, laws of eighteen hundred and eighty-one, as are inconsistent with this act, and all other acts and parts of acts inconsistent with this act are hereby repealed.

SEC. 15. This act shall take effect immediately.

After the reading of the bill it was, on motion, seconded, taken up and considered by sections. A few objections were raised to certain of its provisions, notably that excluding College men from positions on the Board. The questions were well and earnestly discussed, with the result of the final adoption of the bill as printed by a unanimous vote.

